# Methylthioadenosine (MTA) inhibits melanoma cell proliferation and *in vivo *tumor growth

**DOI:** 10.1186/1471-2407-10-265

**Published:** 2010-06-08

**Authors:** Pedro Andreu-Pérez, Javier Hernandez-Losa, Teresa Moliné, Rosa Gil, Judit Grueso, Anna Pujol, Javier Cortés, Matias A Avila, Juan A Recio

**Affiliations:** 1 Medical Oncology Research Program, Vall d'Hebron Research Institute, Vall d'Hebron Institute of Oncology (VHIO) Vall d'Hebron Hospital Barcelona 08035, Spain; 2 Pathology Department, Vall d'Hebron Hospital Barcelona 08035, Spain; 3 Clnical Oncology Department, Vall d'Hebron Hospital, Barcelona 08035, Spain; 4 Division of Hepatology and Gene Therapy, CIMA, University of Navarra, Pamplona, Spain

## Abstract

**Background:**

Melanoma is the most deadly form of skin cancer without effective treatment. Methylthioadenosine (MTA) is a naturally occurring nucleoside with differential effects on normal and transformed cells. MTA has been widely demonstrated to promote anti-proliferative and pro-apoptotic responses in different cell types. In this study we have assessed the therapeutic potential of MTA in melanoma treatment.

**Methods:**

To investigate the therapeutic potential of MTA we performed *in vitro *proliferation and viability assays using six different mouse and human melanoma cell lines wild type for RAS and BRAF or harboring different mutations in RAS pathway. We also have tested its therapeutic capabilities *in vivo *in a xenograft mouse melanoma model and using variety of molecular techniques and tissue culture we investigated its anti-proliferative and pro-apoptotic properties.

**Results:**

*In vitro *experiments showed that MTA treatment inhibited melanoma cell proliferation and viability in a dose dependent manner, where BRAF mutant melanoma cell lines appear to be more sensitive. Importantly, MTA was effective inhibiting *in vivo *tumor growth. The molecular analysis of tumor samples and *in vitro *experiments indicated that MTA induces cytostatic rather than pro-apoptotic effects inhibiting the phosphorylation of Akt and S6 ribosomal protein and inducing the down-regulation of cyclin D1.

**Conclusions:**

MTA inhibits melanoma cell proliferation and *in vivo *tumor growth particularly in BRAF mutant melanoma cells. These data reveal a naturally occurring drug potentially useful for melanoma treatment.

## Background

Melanoma is a common skin cancer resulting in high morbidity and mortality. The development of effective therapeutics designed to target melanoma has become the recent focus of research to improve the melanoma patient's prognosis.

In mammalian cells, 5'-Methylthio-5'deoxyadenosine (MTA) is formed from decarboxylated S-adenosylmethionine in the biosynthesis of spermidine and spermine, and is cleaved by MTA phosphorylase (MTAP) into adenine and 5'-methylthio-5'deoxyribose-1-phospate, which are used for the salvage of ATP and methionine respectively [1].

The *MTAP *gene lies on 9p21, close to the gene *CDKN2A *that encodes the tumor suppressor proteins p16^INK4A ^and p14^ARF ^being widely expressed in normal cells and tissues [2]. The *INK4A-ARF *locus on chromosome 9p21, (encoding p16^INK4a ^and p14^ARF^), is often deleted in human melanoma [3]. Interestingly, *MTAP *and *CDKN2A *are frequently homozygously co-deleted otherwise, inactivated in tumor cells including melanoma [4], resulting in higher intra and extracellular MTA levels [5]. A wide variety of biological responses to MTA have been reported both *in vivo *and in cell culture. While physiological intracellular concentrations of MTA in the nM range might have a tumor-supporting role in MTAP deficient-melanoma cells [5], the administration of higher concentrations of MTA (μM) interfere with cell proliferation, lymphocyte activation, tumor development, invasiveness and the regulation of apoptosis [6-10]. Moreover, it has been shown that MTA has a differential effect in normal and transformed cells. While hepatocarcinoma cells undergo apoptosis when treated with MTA, normal hepatocytes and normal human fibroblast remain viable and are protected from okadaic acid-induced programmed cell death [5,10,11]. Importantly, MTA has been tested in mice and rats and found to be non-toxic at high doses even when given over extended periods [12,13].

Although the mechanisms of action of MTA are not fully understood, it has been postulated that the inhibition of polyamine synthesis could be responsible for the cytostatic effects of MTA. Moreover, MTA has been shown to interfere with key cell signaling pathways, being able to inhibit growth-factor induced protein tyrosine phosphorylation and to increase intracellular cAMP levels through the inhibition of cAMP phos-phodiesterase [14,15]. Additionally, MTA inhibits protein methylation, modulating cell signaling and protein expression [9,16,17].

A number of studies have demonstrated the effects of MTA in tumoral cell lines. However, *in vivo *studies have been restricted to gastroenterological tumor models or brain autoimmune disease. Besides the continuous efforts from the scientific community, there are not effective therapeutic approaches for melanoma treatment. In this study we explored the therapeutic properties of MTA in melanoma treatment. We used several human and mouse melanoma cell lines having different mutational status respect RAS, and BRAF proteins, and investigated the inhibition capabilities of MTA *in vitro*. We also performed *in vivo *studies using a mouse melanoma xenograft model showing the effectiveness of MTA in melanoma treatment.

## Materials and methods

### Cell lines

37-31E mouse melanoma cells were described previously [18-20]. UACC903 cells were a gift from J. Trent (P. Pollock, Tgen, Phoenix, AR, USA). SkMel147 and SKMel103 cells were obtained from M. Soengas (CNIO Madrid, Spain) and MeWo and SKMel28 cells were purchased from the ATCC. 37-31E, SkMel147, SKMel103 and MeWo cells were maintained in DMEM with 10% FBS, penicillin/streptomycin. 37-31E cells were supplemented with EGF (50 ng/ml) (Invitrogen, Carlsbad, CA, USA) and Insulin (4 μg/ml) (Invitrogen, Carlsbad, CA, USA) and grown at 37°C and 5% CO_2 _conditions. UACC903 were maintained in RPMI medium with 10% FBS, penicillin/streptomycin (Invitrogen, Carlsbad, CA, USA).

### Antibodies and Western Blot analysis

Cells were lysed in RIPA buffer containing phosphatase and protease inhibitors (Sigma-Aldrich, Saint Louis, MO, USA). Liquid nitrogen frozen tumor samples were homogenized in RIPA buffer. 50 μg of total protein lysates were separated by SDS-PAGE and transferred to a membrane. After blocking, membranes were blotted against different primary antibodies and developed using horseradish peroxidase linked secondary antibodies and ECL (GE Healthcare, Barcelona, Spain). Cyclin D1 antibody was from Santa Cruz; phospho-Erk1/2 (Thr202/Tyr204), cleaved-caspase-3, p-S6 (Ser235/236), and phospho-Akt (Thr308) antibodies were from Cell Signaling (Danvers, MA USA); p-Bad was from Genscript, Piscataway, NJ, USA, Ki67 was from Master diagnostica, (Granada, Spain) and GAPDH was from Trevigen (Gaitherburg, MD, USA). CD31 antibody was from DAKO (Spremberg, Denmark). The anti-MTAP antibody was the generous gift of Dr. D.A. Carson (University of California San Diego, CA, USA), and was used at a 1:1.000 dilution.

### Proliferation assays

Cells were seeded one day before treatment (50,000 cells per well (37-31E, MeWo, SkMel103, SkMel147, UACC903,) or 75,000 cells per well (Colo829)). Time point treatments were done in triplicates. Number of viable cells at different time points was analyzed by using Guava-Viacount reagent (Guava Technologies, Hayward, CA, USA), in a cell counter (Viacount; Guava Technologies, Hayward, CA, USA).

### Cell cycle analysis

Cells were grown in complete media and treated for 48 h with 10 μM of MTA. Time point treatments were done in triplicates. Then, medium and cells were recollected and after centrifugation, cells were fixed and stained with the Cell Cycle Analysis Guava-Viacount reagent (Guava Technologies, Hayward, CA, USA). Samples were analyzed with the Guava cytometer PCA (Guava Technologies Hayward, CA, USA).

### *In vivo *studies

Five to six month old male FVB/N mice were injected subcutaneously with one million cells in PBS. When tumors reached between 50-100 mm^3^, mice were treated with either DMSO or MTA (96 μmol/kg body weight), a dose selected for its previously shown efficacy and lack of toxicity in other *in vivo *models [13,21]. MTA was prepared from S-adenosylmethionine (Europharma, Madrid, Spain) as described elsewhere [22]. Treatments were done by IP injection daily. Control mice were treated with the same volume of DMSO (100 μl). Tumor size and mice weight was monitored every two days. Tumor volume was calculated with the equation (d^2^*D)*(π/6) (d = small-diameter; D = big-diameter). When mice were sacrificed, tumors were dissected and processed. All the animal procedures have been approved and supervised by the animal care and ethical committee of the Vall d'Hebron Research Institute.

### Immunohistochemistry, Immunofluorescence, TUNEL assay and microvessel density quantification

Paraffin-embedded tumor samples were subjected to immunocytochemistry according to the manufacturer's antibody protocol. Samples were developed either by using secondary antibodies linked to horseradish peroxidase (HRP) and diaminobenzidine (DAB) as a substrate or by immunofluorescence. Tumor samples were used to perform a TUNEL assay as described previously [23]. Apoptosis and proliferating cells were quantified by calculating the average of positive cells in ten fields (10×). For microvessel quantification, tumor samples were stained for CD31. Then, the integrated density of CD31 fluorescence per field (20× magnification = 587590 μm^2^) was measured using ImageJ software (NIH). Ten fields per sample were quantified for a total of 5 DMSO treated tumor samples and 5 MTA treated tumor samples.

### Colony formation assays

Three hundred cells were seeded. Treatments were added next day. Plates were incubated at 37°C and 5% CO_2 _until differences between the treatment conditions were noticeable. Media was changed every 2 days. Plates were washed with PBS, fixed with 4% formaldehyde (Sigma-Aldrich, Saint Louis, MO, USA) in PBS for 10 minutes, and stained with crystal violet. Finally, representative pictures were taken and the number of clones was quantified. At least two biological replicates with three technical replicates each were performed for every cell line.

### Motility assays

For motility assays 5 × 10^4 ^cells were seeded in a 24 multi-well plate trans-wells (Corning). Following treatment cells were washed with 1× PBS and fixed with 1% glutaraldehyde in PBS. Cells were then stained with an aqueous solution of 0.1% crystal violet. After destaining in water, non-migrating cells in the top of the trans-well were removed, and stained migrating cells in the bottom of the trans-well were destaining with PBS containing 0.2% Triton X-100. The O.D. was then measured at 590 nm[24].

### mRNA samples and qRT-PCR

Fresh tumor tissues were disrupted using a rotor-stator homogenizer. mRNAs from tumors and cell lines were purified using RNeasy Kit (Quiagen). Amount and quality of RNA was assessed by spectrometrical measurements. Two hundred ng of RNA per sample were used to obtain cDNA using SuperScript™ III First-Strand Synthesis System for RT-PCR following the manufacturer's recommendations (Invitrogen, Carlsbad, CA USA). qRT-PCR was performed using validated Taqman Probes (VEGF: Mm 01281447_m1, and 18 S RNA: Hs 03003631_g1); (Applied Biosystems, Foster City, CA USA). qRT-PCR was performed according to manufacturers recommendations in a SDS 7900HT System. 18 S RNA was used as an internal control. Results were calculated using ΔΔCt method.

### Statistics

Comparisons protein expression and tumor size among cell lines or treatment groups were done by two-sided *t *test (Microsoft Excel, Microsoft, Redmond, WA, USA). Clonogenic assays were analyzed using Wilcoxon Signed-Rank Test [25](Vassar Stats, Poughkeepsie, NY USA).

## Results

### Methylthioadenosine (MTA) inhibits melanoma cell proliferation

**Figure 1:**
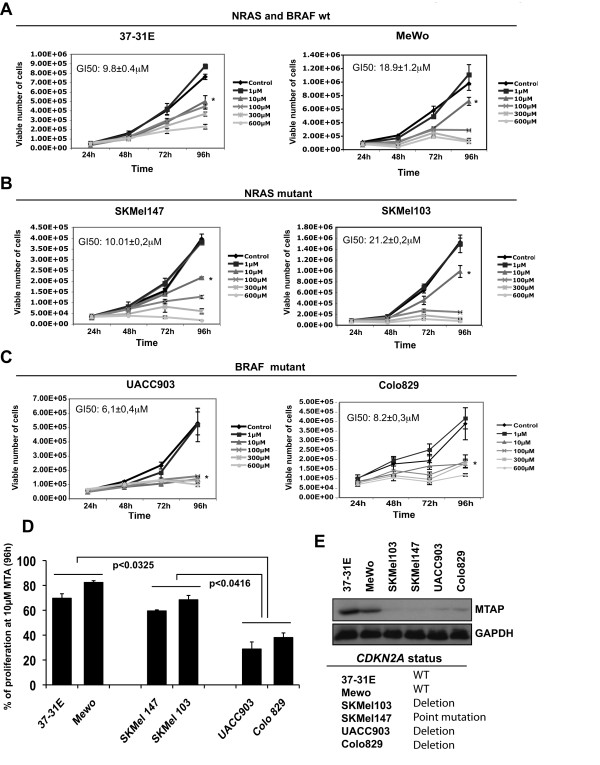
**Methylthioadenosine inhibits melanoma cell growth**. Proliferation assays of (A) 37-31E and MeWo (wild type RAS BRAF), (B) SKMel147 and SKMel103 (NRAS^Q61L ^mutated) and (C) UACC903 and Colo829 (BRAF^V600E ^mutated) melanoma cells in complete medium with increasing concentrations of Methylthioadenosine (MTA). Medium was changed adding fresh MTA every 48 h. Proliferation assays were performed by counting the number of viable cells using Guava-Viacount reagent (Guava Technologies) in a cell counter (Viacount) at the time points indicated. Asterisk indicate p < 0.05. (D) Graph showing the percentage of cell proliferation inhibition at 96 h treated with 10 μM MTA. *p*-values were calculated performing a *t*-student test. (E) Western-blot showing the levels of MTAP in the different cell lines. The mutational status of *CDK2NA *for the different cell lines is showed in the table.

To investigate the inhibition capabilities of methylthioadenosine (MTA) on melanoma cells, we performed cell proliferation assays. Melanoma cell lines harboring wild type NRAS and BRAF (37-31E and MeWo), NRAS^Q61L ^mutation (SKMel 103 and SKMel 147) or BRAF^V600E ^mutation (UACC903, Colo 829) were grown in complete medium and in the presence of increasing concentrations of MTA. Wild type NRAS and BRAF melanoma cells (37-31E, MeWo) (Fig 1A), and melanoma cells harboring NRAS^Q61L ^(SKMel 147 and SKMel 103) (Fig 1B) showed a 50% inhibition of cell proliferation at 9.8 ± 0.4 μM, 18.9 ± 1.2 μM, 10.01 ± 0.2 μM and 21.2 ± 0.2 μM respectively, while the GI50 for BRAF^V600E ^mutant cell lines was 6.1 ± 0.4 for UACC903 cells and 8.2 ± 0.3 for Colo 829 cells (Fig 1C). BRAF^V600E ^mutant cells showed a significant lower proliferation rate (p < 0.05) at 96 h using 10 μM MTA compared with wild type or NRAS^Q61L ^mutant cells (Fig 1D). All melanoma cell lines were significantly (p < 0.01) more sensitive than normal MEFs (GI50>600 μM) (additional file 1, panel A), reaching the maximum inhibition of proliferation between 10 μM and 600 μM concentration of MTA depending on the cell line (Fig 1). Moreover, cells harboring deletions in p16INK4a showed lower levels of MTAP, and 3 out of 4 of these cell lines appeared to be more sensitive to MTA treatment (Fig 1E).

These results indicate that MTA is effective inhibiting *in vitro *melanoma cells proliferation. Interestingly, the results also suggest that BRAF mutant cells and probably cells showing low levels of MTAP would be more sensitive to the MTA treatment.

### MTA reduces melanoma cells survival

**Figure 2:**
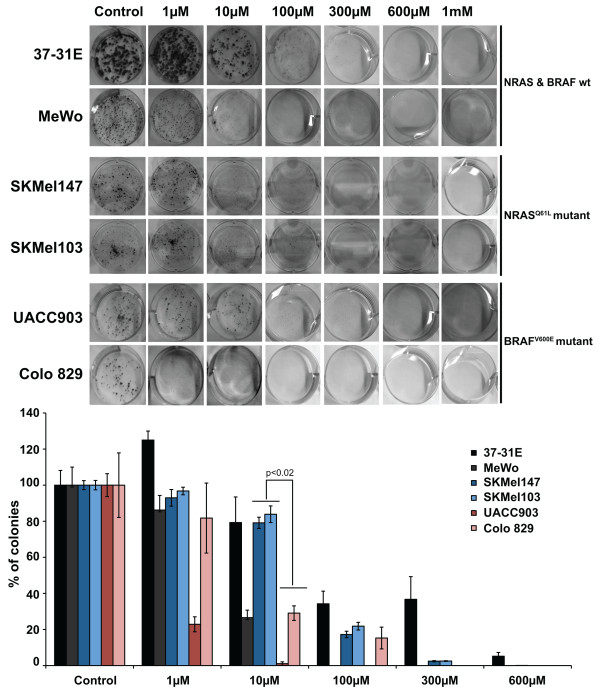
**Methylthioadenosine (MTA) decreases melanoma cell viability**. Clonogenic assays of 37-31E, MeWo, SKMel147, SKMel103, UACC903 and Colo 829 melanoma cells in complete medium with increasing concentrations of Methylthioadenosine (MTA). Three hundred cells were seeded clones were visualized by crystal violet staining and counted. Fresh media was changed every 48 h. Graphs represent the percentage reduction in the number of clones upon different treatments. *p-value *was calculated performing a Wilcoxon-signed rank test (Vassar Stats).

We next investigated whether MTA interferes with melanoma cells survival capabilities. To that end, we performed clonogenic assays in several melanoma cell lines adding increasing concentrations of MTA (1 μM to 1 mM). Interestingly, 1 μM concentration of MTA promoted an increase in the number of colonies in 37-31E cells, with no detectable effects in the other of cell lines tested except for the BRAF^V600E ^mutant cell line Colo829 where 1 μM of MTA totally suppressed the colony formation. At 10 μM of MTA BRAF^V600E ^mutant cell lines were significantly more sensitive (p < 0.02) than RAS^Q61L ^mutant cell lines. The addition of 100 μM of MTA, or higher concentrations, was very effective inhibiting melanoma cell viability, independently of the RAS pathway mutational status (Fig 2). Altogether, the results showed that at 100 μM or higher concentrations of MTA there was not any difference in viability in response to MTA treatment among cell types harboring wild type or mutant RAS pathway. However, at lower concentrations (10 μM) BRAF^V600E ^mutant cells were slightly more sensitive than wild type or RAS^Q61L ^mutant cell lines.

### MTA treatment inhibits melanoma *in vivo *tumor growth

**Figure 3:**
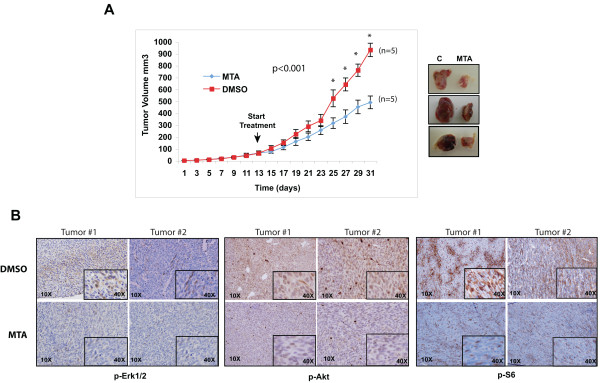
**Methylthioadenosine (MTA) inhibits *in vivo *tumor growth and decreases the activation of RAS and PI3K tumor signaling pathways**. **(A) **37-31E melanoma cell line was subcutaneously injected into immunocompetent FVB/N mice. The animal groups were treated with DMSO (Control) (n = 7) or methylthioadenosine (MTA) (n = 7). Tumor size was calculated as described in materials and methods. *p*-values were calculated performing a *t*-student test. Representative photographs of three paired untreated and MTA-treated excised melanoma tumors are showed on the right. **(B) **Immunostaining of paraffin-embedded tumor sections showing the levels of p-Erk1/2, p-Akt and p-S6 (20×). Insets show a 40× detail of the picture. Representative images from two different untreated and treated tumors are shown.

In view of to the inhibitory capabilities of MTA *in vitro*, we tested whether or not MTA was effective inhibiting *in vivo *tumor growth. To answer this question we performed an *in vivo *experiment in a melanoma xenograft mouse model. The FVB/N syngenic 37-31E melanoma cell line was subcutaneously injected into FVB/N mice. Then, mice were divided in two groups and treated with DMSO (control) and MTA respectively. When tumors reached between 50-100 mm^3 ^the drug was administrated daily via intraperitoneal letting the tumors grow for a total of thirty days. Mice treated with MTA showed a significant decrease (47%) in the tumor volume (p < 0.001) compared with the controls (DMSO) (Fig 3A) with no evident toxic effects according to the mice weight (data not shown) and liver function tests (ALT_(iu/L)_: control: 19 ± 2; MTA: 18 ± 3; AST_(iu/L)_: control: 45 ± 4; MTA: 54 ± 3).

We investigated the status of p-Erk1/2, p-Akt and p-S6 by immunohistochemistry using paraffin-embedded tumor samples. Tumor samples treated with MTA showed a diminished activity of the PI3K and mTOR pathways according to the p-Erk1/2, p-Akt and p-S6 levels (Fig 3B).

Altogether, these results indicate that MTA treatment is effective blocking melanoma *in vivo *tumor growth interfering with the activation of PI3K and mTOR pathways.

### MTA promotes cytostatic effects rather than pro-apoptotic responses

Several publications have demonstrated both the cytostatic and pro-apoptotic effects of MTA in mammalian cells [10,26-28]. In our system, the proliferation curves and *in vivo *results suggested that MTA might be inhibiting cell proliferation and *in vivo *tumor growth through cytostatic rather than pro-apoptotic mechanisms. To further confirm this hypothesis we analyzed proliferation markers and the apoptosis levels of the tumor samples and performed *in vitro *experiments to investigate the effects of MTA in promoting apoptosis.

**Figure 4:**
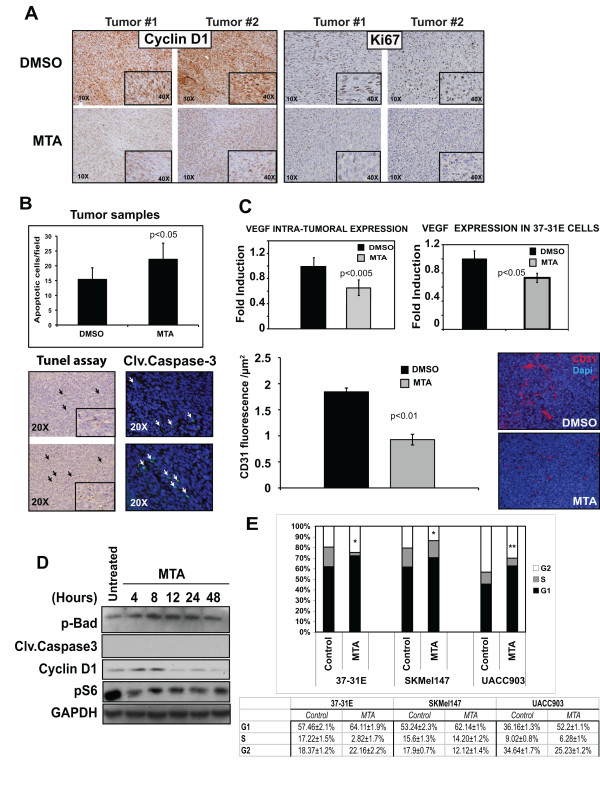
**Methylthioadenosine (MTA) promotes cytostatic effects rather than pro-apoptotic effects in melanoma tumors and cells**. **(A) **Tumor sections from mice treated either with DMSO or MTA were stained with cyclin D1 and Ki67 antibodies. Representative pictures are shown. **(B) **Quantification of apoptotic cells within the tumors. Paraffin-embedded tumor samples were subjected to TUNEL assay or stained against cleaved caspase-3. Graph shows the quantification of the TUNEL assay. Positive cells from ten fields (20×) per sample were quantified and the average number of cells per field was calculated. **(C) **Upper graph, quantification by qRT-PCR of VEGF levels in tumor samples. Lower graph, qRT-PCR of VEGF expression levels. 37-31E were untreated (Control) or treated for 48 h with 10 μM of MTA in complete medium. Microvessel's density quantification in xenografts. Graph shows CD31 fluorescence per μm^2^. Representative pictures are showed on the right. *p*-values were calculated performing a *t*-student test. **(D) **37-31E cells were treated with MTA (10 μM) for the time points indicated. Fifty micrograms of total lysates were resolved by PAGE-SDS. p-Bad, cleaved-caspase3, p-S6 and cyclin D1 protein levels are showed. GAPDH is used as a loading control. **(E) **MTA treatment induces a slowdown cell cycle G1 phase. Cells were grown in complete medium for 48 h in the presence or absence of MTA (10 μM). Cell cycle analysis was measured in triplicates using Cell Cycle Analysis Guava-Viacount reagent (Guava Technologies). Average of the three samples in each phase of the cell cycle are shown. *p*-values were calculated performing a *t*-student test (* = p < 0.05; ** = p < 0.01).

Immunostaining of the tumor tissues showed that samples from MTA treated mice had lower levels of cyclin D1 expression that closely correlated with a lower tumor proliferation index according to the Ki67 immunostaining (Fig 4A). Apoptosis within the tumor was measured by TUNEL assay and quantification of cleaved-caspase-3 positive tumor cells in paraffin sections. Our results showed that MTA-treated samples had slightly higher levels of apoptotic cells compared with control tumors (Fig 4B). In addition to this, it is known that angiogenesis is directly related to tumor growth. Interestingly, the intra-tumoral expression of VEGF, as well as the basal expression of VEGF in 37-31E cells was down regulated by MTA treatment (Fig 4C). Furthermore, these data correlated with a significant lower vessel density of MTA-treated tumor samples according to CD31 staining (Fig 4C).

We further analyzed the effect of MTA *in vitro*. 37-31E cells were treated with 10 μM MTA for different periods of time up to 48 h. At the molecular level the results showed that p-Bad and cleaved-caspase-3 levels did not change significantly over time, indicating the low levels of apoptosis (Fig 4D). However, MTA treatment promoted the de-phosphorylation of the mTOR downstream target ribosomal protein S6 and the down-regulation of the cyclin D1 protein levels, supporting the cytostatic effects of MTA (Fig 4D). Additionally, motility of 37-31E cells was also significantly reduced upon MTA treatment (additional file 1, panel B). Importantly, cell cycle analysis of melanoma cells harboring wild type RAS and BRAF (37-31E), NRAS^Q61L ^mutation (SKMel147) and BRAF^V600E ^mutation (UACC903) treated with 10 μM MTA for 48 h, showed an increased number of cells in G1 phase compared with untreated cells (6.6% ± 0.8% (p < 0.05), 8.9% ± 0.5% (p < 0.05) and 16.4% ± 0.7% (p < 0.01) respectively) (Fig 4E). Interestingly BRAF^V600E ^mutant cells UACC903 showed the highest proportion of cells in G1 phase, indicating a slowed down cell cycle

Altogether, these results show evidences indicating that cytostatic effects on tumor cells mediate the reduction in melanoma tumor growth upon MTA treatment.

## Discussion

Melanoma is the most serious form of skin cancer. If it is not recognized and treated early, the cancer can advance and spread to other parts of the body, where it becomes hard to treat and can be fatal. Recent studies have provided a much-improved understanding of melanoma biology, however, this knowledge has yet to be translated into effective treatment strategies. In this study, we investigated the therapeutic capabilities of MTA in melanoma treatment, a natural occurring nucleoside that has been shown to be effective in other tumor types [6,10,16,29,30]. Our results show that MTA inhibits *in vitro *cell proliferation, and viability in a dose dependent manner in a variety of human and mouse melanoma cell lines. Importantly, MTA treatment was also effective inhibiting *in vivo *tumor growth in a mouse melanoma xenograft model. Furthermore, the molecular analysis of the tumor samples and experiments performed with the cell lines indicated that in our model MTA has mostly cytostatic rather that pro-apoptotic effects.

Besides the inhibitory effects on the polyamine biosynthesis, MTA has been shown to exert other potent and specific pharmacological effects on cellular functions such as proliferation, apoptosis and modulation of the immune system [10,31,32]. Our current findings are in agreement with previous publications showing the inhibitory effects of MTA on proliferation and invasion (Fig 1 and additional file 1) of different types of tumor cells lines [33-35]. It is known that genetic mutations within tumor cells condition the drug response and this could be taken as an advantage in the design of more effective therapeutic approaches. Interestingly, BRAF mutant melanoma cell lines (UACC903 and Colo829) showed the highest sensitivity to MTA treatment, where concentrations of 10 μM of MTA reduced proliferation up to 70% (Fig 1D). Why BRAF mutant cells are more sensitive to MTA treatment is unknown and is an area of our current investigation. Nevertheless, the inhibition of cyclin D1 and proliferation by MTA might have a more pronounced effect in cells addicted to oncogenes with potent mitogenic effects. Additionally, the low levels of MTAP protein expressed in both cell lines might be contributing to the observed response.

In agreement with the *in vitro *results, MTA treatment reduced *in vivo *tumor growth by 45%. The molecular analysis of the tumors indicated that MTA treated tumors had lower rates of proliferation according to the Ki67 and cyclin D1 levels, that correlated with lower levels in the PI3K and mTOR pathway activation and VEGF expression. While several studies have shown pro-apoptotic effects of MTA in tumor treatment [10,31], we observed a small increase of apoptosis in our tumor samples.

Recently, it has been described that MTA was able to induce the expression of growth factors and matrix metalloproteases in melanoma cells as well as enhance invasion and vasculogenic mimicry [5]. It is widely known that MTA inhibits methyltransferase enzymatic reactions and interferes with cellular pathways modulating cell signaling and protein expression [5,14,16,17]. In the mentioned study, the obtained data was generated at early time points of MTA treatment. Furthermore, the biological outcome in a long-term treatment, such as melanoma cell proliferation and the *in vivo *melanoma tumor growth, were not assessed. Our data show that the inhibitory effects of MTA on melanoma cell proliferation occur mostly after 48 h treatment. We believe that the biological response to MTA is dose dependent and cell type dependent. Indeed, several publications have shown that fibroblasts and normal hepatocytes have contrary responses to MTA compared to tumor cells. Moreover, in our system low concentrations of MTA (1 μM) promoted a slight increase in proliferation and viability, supporting a possible cell-type specific differential response to low concentrations of MTA. In mice, after intraperitoneal administration at 75 mg/kg, serum levels of MTA rapidly reached a peak of 28 μM rapidly and, at 30 minutes MTA was still at 10 μM [12]. In our hands a preliminary study of the bioavailability of MTA showed that plasma concentrations 20 min after i.p administration of this compound at 96 μmol/kg (equivalent to 30 mg/kg) were in the micromolar range (20-30 μM) (unpublished observations). In view of the efficacy of MTA reducing *in vivo *tumor growth, we speculate that the concentration reached within the tumor should be higher than 1 μM.

Previous studies have described a cytostatic effect of MTA on Mewo-LC1, Raji and R1.1 H cells [26-28]. According to our current observations in the xenograft model, MTA promoted cytostatic rather than pro-apoptotic effects. This result was confirmed by the *in vitro *experiments using the same cell line, where we found that MTA treatment induced the dephosphorylation of the downstream mTOR target S6 ribosomal protein, and the decrease of cyclin D1 protein levels. Importantly, we did not observe any molecular indication of apoptosis. Supporting these results, treatment of melanoma cells with MTA induced a cell cycle slow down in G1 phase. Interestingly, in agreement with the proliferation and viability results, UACC903 BRAF mutant melanoma cells showed the higher accumulation of cells in G1 phase.

MTA is a well-tolerated drug, devoid of the unwanted effects of other methyltransferase inhibitors. It has been administered previously in both acute and chronic experimental models of liver injury and systemic inflammation, showing efficacy and a safe profile [32] with an ID50 of 2.9-0.4 gm/kg (intramuscular) in rats [13]. In humans, MTA is also well tolerated [34,35]. Thus, MTA or any of its synthesized analogs would be good candidates for melanoma treatment in patients

## Conclusions

Altogether, here we show the therapeutic potential of the naturally occurring nucleoside MTA in melanoma treatment. Importantly, our results demonstrate that MTA inhibits melanoma cell proliferation and *in vivo *tumor growth, supporting the antitumoral potential of MTA shown by others in other type of tumors. The data also suggest that BRAF^V600E ^mutant cells appeared to be more sensitive to the cytostatic effects mediated by MTA. This study outlines an efficacious and well-tolerated therapeutic candidate for melanoma intervention.

## Competing interests

The authors declare that they have no competing interests.

## Authors' contributions

PAP did most of the experimental work and data analysis. AP was involved in the animal work. JG She was involved in the experimental work. JHL, RG and TM performed the pathological and immunohistochemical analysis. JC was involved in the analysis and discussion of the data. MAA contributed with some reagents, analyzed and discussed the data. JAR conceived and designed the study, coordinated the work, anlayzed the data and wrote the paper. All the authors approved and read the final manuscript.

## Pre-publication history

The pre-publication history for this paper can be accessed here:

http://www.biomedcentral.com/1471-2407/10/265/prepub

## Supplementary Material

Additional file 1 **Figure S1**. **A) MTA does not inhibit proliferation and viability of normal cells (MEFs)**. Cell proliferation of mouse embryo fibroblasts (MEFs) in response to increasing concentration of MTA. Medium was changed adding fresh MTA every 48 h. Proliferation assays were performed by counting the number of viable cells using Guava-Viacount reagent (Guava Technologies) in a cell counter (Viacount) at the time points indicated. Clonogenic assays using MEFs in complete medium with increasing concentrations of Methylthioadenosine (MTA). Three hundred cells were seeded. Clones were visualized by crystal violet staining and counted. Fresh media was changed every 48 h. Graphs represent the percentage reduction in the number of clones upon different treatments. p-value was calculated performing a Wilcoxon-signed rank test (Vassar Stats). **(B) MTA inhibits motility of 37-31E melanoma cells: **37-31 melanoma cells were seeded in a 24 multi-well plate trans-wells (Corning). Following treatment (either DMSO or MTA 100 μM for the times indicated) cells were washed with PBS and fixed with 1% glutaraldehyde in PBS. Cells were then stained with an aqueous solution of 0.1% crystal violet. After destaining in water, non-migrating cells in the top of the trans-well were removed, and stained migrating cells in the bottom of the trans-well were destaining with PBS containing 0.2% Triton X-100. The O.D. was then measured at 590 nm (Gillies et al., 1986). All the experiments were done in triplicates. p-values were calculated performing a t-student test (*) = p < 0.01. Click here for file
