# Supplementary figures and images for: Methylthioadenosine (MTA) inhibits melanoma cell proliferation and in vivo tumor growth
Source: BMC Cancer. 2010 Jun 8;10:265. doi: 10.1186/1471-2407-10-265 (PMC2891639; doi:10.1186/1471-2407-10-265)

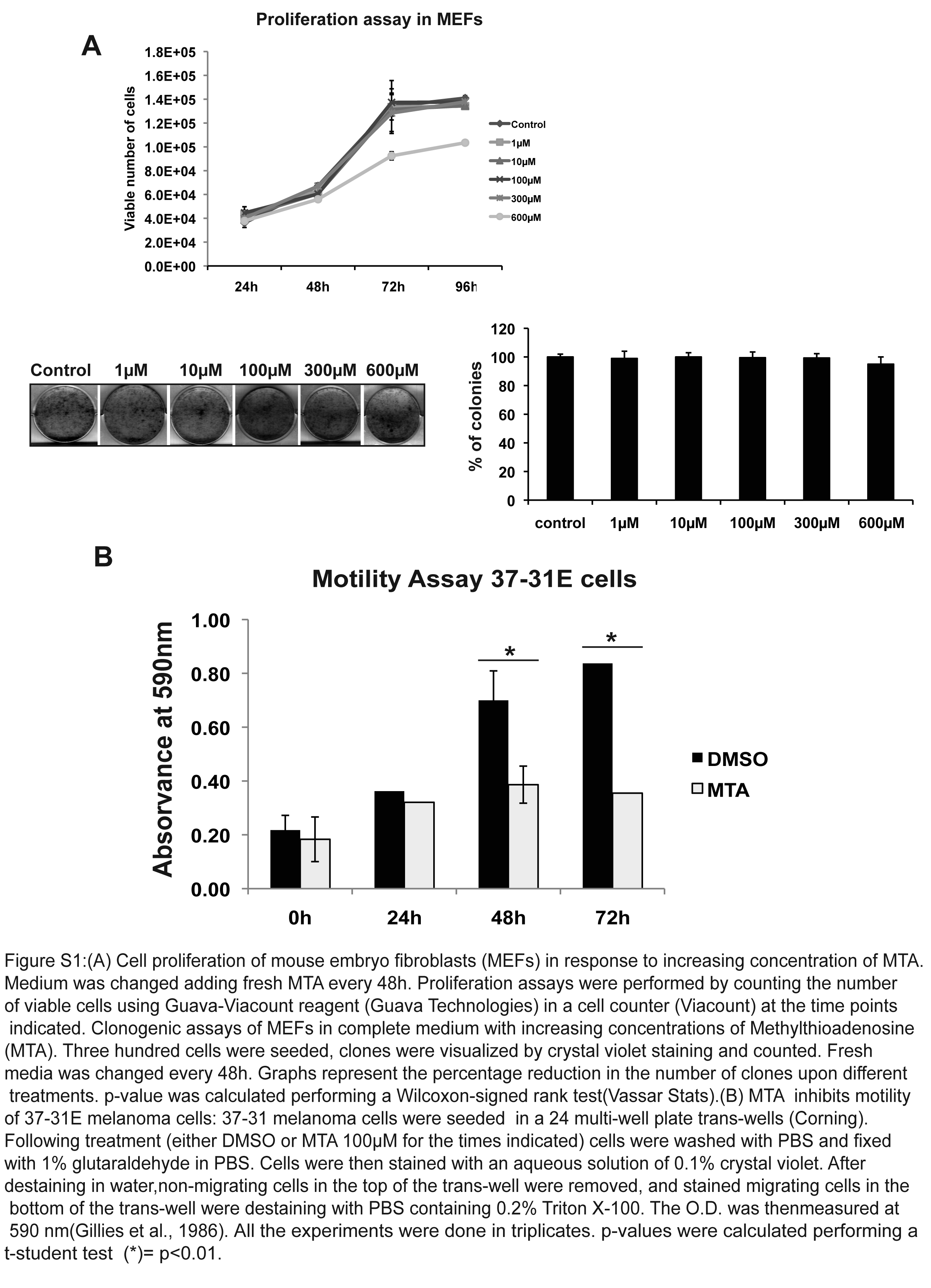

Supplement: Additional file 1 — Figure S1. A) MTA does not inhibit proliferation and viability of normal cells (MEFs). Cell proliferation of mouse embryo fibroblasts (MEFs) in response to increasing concentration of MTA. Medium was changed adding fresh MTA every 48 h. Proliferation assays were performed by counting the number of viable cells using Guava-Viacount reagent (Guava Technologies) in a cell counter (Viacount) at the time points indicated. Clonogenic assays using MEFs in complete medium with increasing concentrations of Methylthioadenosine (MTA). Three hundred cells were seeded. Clones were visualized by crystal violet staining and counted. Fresh media was changed every 48 h. Graphs represent the percentage reduction in the number of clones upon different treatments. p-value was calculated performing a Wilcoxon-signed rank test (Vassar Stats). (B) MTA inhibits motility of 37-31E melanoma cells: 37-31 melanoma cells were seeded in a 24 multi-well plate trans-wells (Corning). Following treatment (either DMSO or MTA 100 μM for the times indicated) cells were washed with PBS and fixed with 1% glutaraldehyde in PBS. Cells were then stained with an aqueous solution of 0.1% crystal violet. After destaining in water, non-migrating cells in the top of the trans-well were removed, and stained migrating cells in the bottom of the trans-well were destaining with PBS containing 0.2% Triton X-100. The O.D. was then measured at 590 nm (Gillies et al., 1986). All the experiments were done in triplicates. p-values were calculated performing a t-student test (*) = p < 0.01. [file 1471-2407-10-265-S1.PNG]
